# Effects of femtosecond laser and other surface treatments on the bond strength of metallic and ceramic orthodontic brackets to zirconia

**DOI:** 10.1371/journal.pone.0186796

**Published:** 2017-10-19

**Authors:** Verónica García-Sanz, Vanessa Paredes-Gallardo, Carlos Bellot-Arcís, Omel Mendoza-Yero, Carlos Doñate-Buendía, Javier Montero, Alberto Albaladejo

**Affiliations:** 1 Orthodontics Teaching Unit, Department of Stomatology, Faculty of Medicine and Dentistry, University of Valencia. Valencia, Spain; 2 GROC•UJI, Institute of New Imaging Technologies, Universitat Jaume I, Castellón, Spain; 3 Orthodontics Teaching Unit, Department of Surgery, Faculty of Medicine, University of Salamanca, Salamanca, Spain; VIT University, INDIA

## Abstract

Femtosecond laser has been proposed as a method for conditioning zirconia surfaces to boost bond strength. However, metallic or ceramic bracket bonding to femtosecond laser-treated zirconia surfaces has not been tested. This study compared the effects of four conditioning techniques, including femtosecond laser irradiation, on shear bond strength (SBS) of metallic and ceramic brackets to zirconia.Three hundred zirconia plates were divided into five groups: 1) control (C); 2) sandblasting (APA); 3) silica coating and silane (SC); 4) femtosecond laser (FS); 5) sandblasting followed by femtosecond laser (APA+SC). A thermal imaging camera measured temperature changes in the zirconia during irradiation. Each group was divided into 2 subgroups (metallic vs ceramic brackets). SBS was evaluated using a universal testing machine. The adhesive remnant index (ARI) was registered and surfaces were observed under SEM. Surface treatment and bracket type significantly affected the bracket-zirconia bond strength. SBS was significantly higher (p<0.001) for ceramic brackets in all groups (APA+FS > APA > FS > SC > control) than metallic brackets (APA+FS > FS > SC > APA > control). For metallic brackets, groups SC (5.99 ± 1.86 MPa), FS (6.72 ± 2.30 MPa) and APA+FS (7.22 ± 2.73 MPa) reported significantly higher bond strengths than other groups (p < 0.05). For ceramic brackets, the highest bond strength values were obtained in groups APA (25.01 ± 4.45 MPa), FS (23.18 ± 6.51 MPa) and APA+FS (29.22 ± 8.20 MPa).Femtosecond laser enhances bond strength of ceramic and metallic brackets to zirconia. Ceramic brackets provide significantly stronger adhesion than metallic brackets regardless of the surface treatment method.

## Introduction

With the introduction of ceramic esthetic brackets, recent years have seen increased demand for orthodontic treatments that minimize the visual impact of the apparatus [1]. As a consequence, bonding these appliances to different surfaces such as ceramic, has gained clinical relevance as many adult patients have ceramic dental restorations such as crowns or bridge-work [2].

Dental ceramics, especially zirconia, are excellent materials for dental restoration, and bonding to these materials has been widely studied [3–11].

Due to the properties of these ceramic materials, bonding brackets to their surfaces can be complicated [4]. For this reason, it is necessary to determine a bonding protocol that is available to all clinicians, and will achieve efficient and durable bracket-zirconia bonding.

The surface conditioning techniques commonly used for zirconia bonding are: sandblasting [3, 5]; silica coating [6]; etching with hydrofluoric acid [7]; laser irradiation with CO_2_ or Er:YAG [8–11]. However, an ideal zirconia surface treatment–one that will provide sufficient bond strength to minimize bracket debonding from zirconia surfaces–has not yet been established.

Femtosecond lasers have been proposed as an alternative for treating zirconia surfaces in an attempt to improve the adhesion of dental cements and orthodontic brackets [12–15]. These lasers, consisting of a Titanium-Sapphire oscillator, provide ultrashort pulses in the femtosecond range, and cause no thermal damage to the irradiated surfaces [16].

Only two studies have analyzed the shear bond strength of brackets bonded to ceramic surfaces treated with femtosecond laser [12, 13]. In both investigations, the authors used metallic brackets; no study has ever assayed the performance of ceramic brackets bonded to femtosecond laser-treated porcelain surfaces. To the authors’ knowledge, only one study has compared the shear bond strength of metallic brackets in comparison with ceramic brackets bonded to ceramic surfaces [17]. Testing the differences between these interfaces is of clinical relevance, given the high demand for aesthetic orthodontic treatments by adult patients with ceramic restorations. There is a clear need to determine the most efficient method of treating zirconia surfaces for optimal ceramic and metallic bracket bonding.

The aim of this study was to compare the effect of four different zirconia conditioning techniques (air particle abrasion, silica coating, femtosecond laser irradiation, and air particle abrasion followed by femtosecond laser irradiation) on the shear bond strength of metallic and ceramic orthodontic brackets bonded to zirconia surfaces. The null hypothesis was that neither the ceramic surface conditioning technique nor the bracket type would affect the bracket-zirconia shear bond strength.

## Materials and methods

### Sample preparation

Three hundred square densely sintered Yttria Tetragonal Zirconia Polycrystal (Y-TZP) (Cercon®, DeguDent, Hanau, Germany) specimens measuring 9 x 9 x 1 mm were used for this in vitro study. The surfaces were wet-polished with 600-grit silicon carbide paper (CUMI, Carborundum Universal Ltd., Chennai, India). Zirconia samples were randomly assigned to five experimental groups (n = 60).

Group 1 (Control): No surface treatment was applied.

Group 2 (Airbone Particle Abrasion, APA): Surfaces were blasted with alumina particles (Al_2_O_3_) (Aquacut, Medivance Instruments Ltd, London, UK) with an average size of 25 µm under a pressure of 0.25 MPa for about 20 sec at a perpendicular distance of 10 mm from the holder.

Group 3 (Silica coating): Surfaces were treated with tribochemical silica coating (30 µm silica particles) applied perpendicularly for 20 sec, at a working distance of 10 mm and a pressure of 0.25 MPa using the Cojet® System (3M ESPE, Seefeld, Germany). Silanization was performed before bonding by applying a uniform layer of Rely X™ ceramic primer (3M Espe, Seefeld, Germany) to the specimen using a mini-sponge and blowing oil-free air across the surface until dry, following the manufacturer’s instructions.

Group 4 (Femtosecond laser irradiation): zirconia surfaces were irradiated with a femtosecond Ti:Sapphire laser (Femtopower Compact Pro–serial number 1046 –, Spectra Physics, Santa Clara, Ca, USA) with a pulse width of 30 fs, full width at half maximum (FWHM) at a central wavelength of 800 nm, a repetition rate of 1 kHz, and an output power of 200mW for 12 minutes. A programmable acousto-optic filter (Dazzler, Fastlite, Valbonne, France) was used to ensure the time compression of laser pulses at the interaction spot between zirconia samples and laser radiation. To obtain the optimal performance for promoting ablation on the zirconia surfaces, the incoming laser beam with a 6mm diameter at the 1/e^2^ point was focused onto the sample surfaces with a 75 mm plano-convex lens. The samples were placed on the surface of a 2D motion controlled stage moving at a constant speed of 1.44 mm/s in the plane of the laser beam focus. A stair-like pattern was carved, the inter-groove distance being 60 μm.

Group 5 (Airbone Particle Abrasion + Femtosecond laser irradiation): Surfaces were sandblasted following the protocol applied in Group 2 followed by laser irradiation using the parameters described for Group 4.

### Temperature measuring

A thermal imaging camera FLYR E60 (FLYR Systems, Wilsonville, OR, USA) was used to measure temperature changes in the zirconia during sample irradiation with femtosecond laser. The camera was mounted on a tripod perpendicular to the sample at a distance of 15 cm. The thermogram recordings were started 2 seconds before irradiation and continued until 2 seconds following its completion.

### Bonding procedure

After preparing the zirconia samples with the different surface treatments, each group was divided into 2 subgroups (n = 30):

Subgroup 1 (Metal bracket): Upper central incisor stainless steel brackets (Victory 3M Unitek, Monrovia, Calif, USA) measuring 3x4 mm, were bonded to the prepared surfaces by a single clinician using the total etch adhesive system consisting of a primer applied to the ceramic surface and an orthodontic adhesive resin applied to the bracket base (Transbond TM XT; 3M-Unitek) following the manufacturer’s instructions. The adhesive layer was polymerized with a light curing unit (XL 3000, 3M ESPE) at 500 mW/cm^2^ intensity, which was applied to the bracket-zirconia sample from the occlusal and gingival bracket edges for 20 seconds.

Subgroup 2 (Ceramic bracket): Upper central incisor polycrystalline alumina brackets (Clarity Advanced 3M Unitek, Monrovia, Calif, USA) were bonded to the prepared surfaces using the same adhesive system as in subgroup 1.

### Shear bond strength test

SBS tests were conducted according to the standards used in the last published studies on bracket-to-ceramic adhesion [12, 13, 17, 18]. All bonded specimens were mounted perpendicularly on acrylic resin bases and underwent shear loading using a knife edge system at a crosshead speed of 0.5 mm / min until they fractured, using a universal testing machine (AGS-X Autograph, Shimadzu Corporation, Kyoto, Japan).

Bond strength values were calculated in MPa by dividing the maximum load recorded on failure (Newtons, N) to the bracket area.

### Bond failure analysis

After debonding, the zirconia surfaces were evaluated using an Axio M1 light microscope (Carl Zeiss, Oberkochen, Germany) at 40× magnifications to assess the failure mode. The adhesive remnant index (ARI), proposed by Årtun and Bergland [19], was used to classify each failure as one of four categories: 0) No adhesive left on the ceramic surface; 1) less of half of the adhesive left, 2) more than half of the adhesive left; 3) All the adhesive left on the surface, with distinct impression of the bracket mesh.

### Scanning Electron Microscope (SEM) examination

Five additional samples in each experimental group were prepared for SEM qualitative analysis (JEOL-JSM-7001F, JEOL Ltd., Tokyo, Japan) at 600× magnification to assess the differences between the surfaces after each conditioning technique.

SEM, at 300x magnification was also used to analyze the surfaces of representative samples after debonding in order to compare morphological differences between experimental groups.

### Statistical analysis

Data were analyzed using SPSS v.16 software (Statistical Package for the Social Sciences, Chicago, IL, USA).

Descriptive statistics, including the mean, standard deviation (SD), median, minimum and maximum SBS (MPa) were calculated; 95% confidence intervals were also included. Homogeneity of the data was evaluated using the Levene test.

Two-way analysis of variance (ANOVA), and Tamhane’s T2 multiple comparison test were used to determine the statistical significance of the differences in mean variables between the five groups. Statistical significance was set at p<0.05.

Lastly, Kruskal-Wallis and multiple Mann-Whitney tests applying Bonferroni correction were used to assess the homogeneity of ARI index data between groups.

## Results

### Temperature

No temperature changes were observed for any of the samples while irradiating the zirconia surfaces with femtosecond laser.

### Shear bond strength (SBS)

SBS values (MPa) for all subgroups are shown in Table 1. Homogeneity of the data was not significant (p < 0.001). Surface conditioning technique and bracket type significantly affected the bracket-zirconia bond strength (Table 2). The SBS results obtained for subgroups of ceramic bracket were notably greater (23.82 ± 6.67) than those obtained for metallic brackets (5.73 ± 2.24), with statistically significant differences in all the surface treatment groups (p<0.001).

**Table 1:** SBS values and standard deviations (MPa) for each experimental subgroup.

	EXPERIMENTAL GROUPS									
	CONTROL		APA Al_2_O_3_		Silica Coating		FS Laser		APA +FS LASER	
	Metal	Ceram	Metal	Ceram	Metal	Ceram	Metal	Ceram	Metal	Ceram
**N**	**30**	**30**	**30**	**30**	**30**	**30**	**30**	**30**	**30**	**30**
**Mean (MPa)**	4.23	20.06	4.46	25.01	5.99	21.62	6.72	23.18	7.22	29.22
**SD**	0.89	2.34	1.21	4.45	1.86	6.48	2.30	6.51	2.73	8.20
*	**e**	**c**	**e**	**ab**	**d**	**bc**	**d**	**abc**	**d**	**a**

* values with the same letter are not statistically different (p>0.05)

**Table 2:** Two-way analysis of variance for shear bond strength results.

Source	Type III Sum of Squares	df	Mean Square	F	p-value
**Corrected Model**	26260,046	9	2917,783	149,457	,000
**Intercept**	65455,294	1	65455,294	3352,797	,000
**Bracket**	24547,461	1	24547,461	1257,387	,000
**Surface treatment**	1184,958	4	296,239	15,174	,000
Bracket * Surface treatment	527,627	4	131,907	6,757	,000
**Error**	5661,551	290	19,523		
**Total**	97376,891	300			
**Corrected Total**	31921,597	299			

1,00 R Squared =, 823 (Adjusted R Squared =, 817)

The shear bond strength of metallic brackets to control and air-particle-abraded specimens was similar (p = 1.000) and significantly lower than other treatment groups (p<0.001). Statistically significant differences were not found between silica coating, FS laser and APA+FS laser groups (p>0.8); the APA+FS laser group obtained the highest SBS values.

For ceramic brackets, the highest SBS was also obtained in the APA+FS laser group, but with no significant differences in comparison with APA and FS laser groups (p>0.1).

### Failure mode analysis

Table 3 shows bond failure type (n and %) for all subgroups. ARI types 2 and 3 were observed for most samples in the silica coating, FS laser and APA+FS laser surface treatment groups, while none of the samples in the control group showed these failure types. For APA specimens, more than 40% of the samples in the metallic bracket subgroup showed type 2 and 0% type 3; for the APA ceramic bracket subgroup, more than 50% of the specimens showed failure type 2. Statistically significant differences were not found between FS laser and APA+FS laser groups, but these groups obtained significant differences in comparison with control and APA groups.

**Table 3:** Bond failure mode results (ARI) (n and %).

EXPERIMENTAL GROUPS																				
	Control				APA Al_2_O_3_				Silica Coating				FS Laser				APA + FS Laser			
	Metal		Ceram		Metal		Ceram		Metal		Ceram		Metal		Ceram		Metal		Ceram	
	N	%	N	%	N	%	N	%	N	%	N	%	N	%	N	%	N	%	N	%
Type 0	21	70	22	73.3	10	33.3	3	10	8	26.7	1	3.3	0	0	0	0	1	3.3	0	0
Type 1	9	30	8	26.7	13	43.3	4	13.3	5	16.7	7	23.3	2	6.7	3	10	7	23.3	2	6.7
Type 2	0	0	0	0	7	23.3	16	53.3	10	33.3	9	30	6	20	7	23.3	12	40	6	20
Type 3	0	0	0	0	0	0	7	23.3	7	23.3	13	43.3	22	73.3	20	66.7	10	33.3	22	73.3
*	e		e		de		bc		bcd		ac		a		a		ab		a	

ARI types: 0) No adhesive left on the surface; 1) less of half of the adhesive left, 2) more than half of the adhesive left; 3) All the adhesive left on the surface, with distinct impression of the bracket mesh.

* values with the same letter are not statistically different (p>0.05)

### SEM analysis

**Fig 1:**
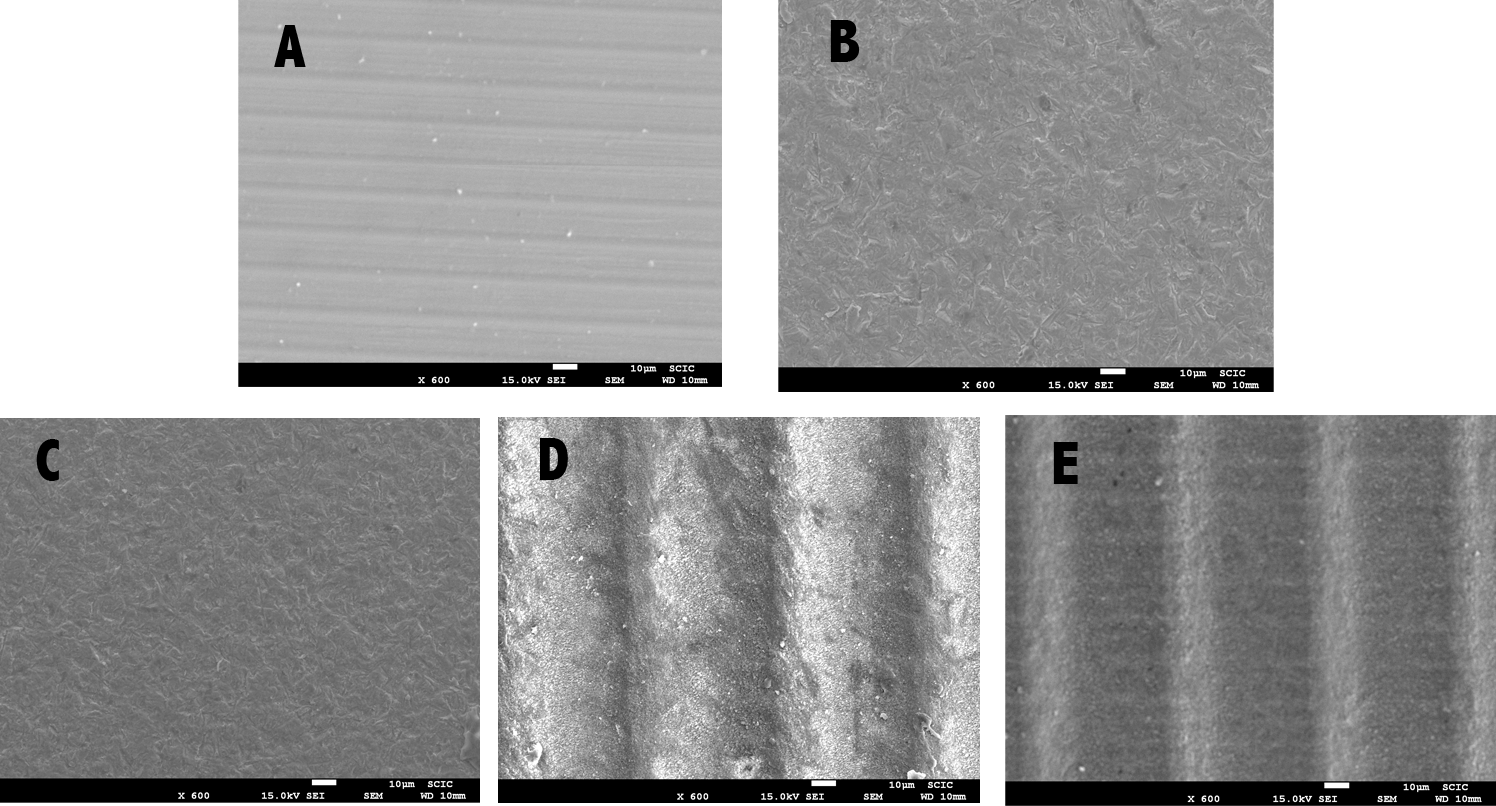
SEM images of zirconia after surface conditioning, at 600× magnification. A = control; B = APA; C = Silica coating; D = FS laser; E = APA+FS laser.

**Fig 2:**
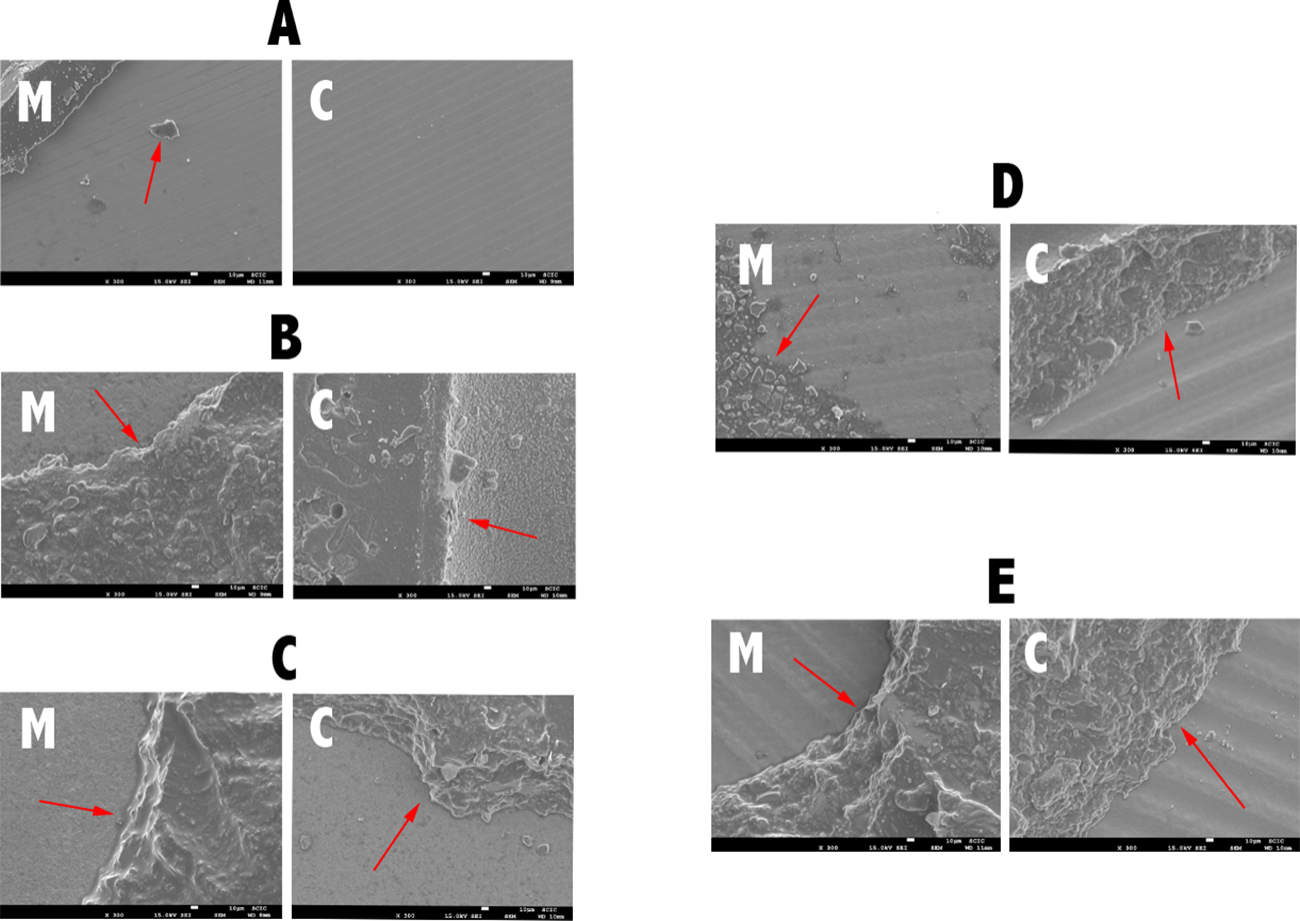
SEM images of zirconia after debonding metal (M) and ceramic (C) brackets, at 300× magnification. A = control; B = APA; C = Silica coating; D = FS laser; E = APA+FS laser.

SEM images of the zirconia surfaces treated with the five different methods are shown in Fig 1. The control group specimen (A) shows a smooth surface with some traces deriving from the polishing procedure, while the specimens from the other groups show different surface morphologies. Some surface roughness can be observed on the APA (B) and silica coating (C) specimens, with a granulated texture. Both femtosecond laser specimens (D and E) show well-defined patterns of parallel grooves. In addition, the APA + FS laser specimen (E) showed a flatter appearance. Fig 2 shows SEM qualitative analysis of representative samples after debonding metal brackets (M) and ceramic brackets (C) from the zirconia surfaces. The control group specimen (A) shows very small amount of adhesive material on the ceramic surface, while other groups show greater amounts of remnant composite resin.

## Discussion

The present study investigated the effects of femtosecond laser irradiation on the shear bond strength of both metallic and ceramic brackets bonded to zirconia surfaces, comparing this surface treatment with other treatments: air-particle abrasion and silica coating.

From the results of this study, the null hypothesis was rejected since significant differences were found between zirconia conditioning techniques groups and bracket type subgroups.

Zirconia flat plates were used so the shape and dimensions of the adherent surfaces could be standardized through the experiment, being reproducible, and so the results could be compared to similar researches [12, 13, 15]. Square shape was chosen rather than round since it was found to be more stable in the acrylic resin bases during testing. Upper central incisor brackets were selected to conduct the study since these brackets have flatter bases than the brackets belonging to the other teeth, thus adapting better to the zirconia surfaces and also allowing more reliable comparisons with other author’s results [13]. These brackets have a rectangular shape with the gingival edge being slightly curved.

Our results showed higher bond strength values for aesthetic ceramic brackets compared with metallic brackets in all treatment groups, with statistically significant differences (p<0.001). To our knowledge, only one work has studied differences in SBS when bonding to ceramic materials and comparing metallic and ceramic brackets, which found no significant differences between ceramic and metallic brackets, contrarily to our study, being the possible reason for this discrepancy between both studies the differences in the ceramic bracket, since they are made of different materials (alumina vs. zirconia) and the bracket bases have different designs and mesh patterns [17].

In this regard, the present study found that adhesive failure (ARI) between the zirconia and the adhesive layer (ARI scores 0 and 1) was, on average, more frequent for the metallic brackets subgroups than for ceramic brackets subgroups.

Ceramic brackets show a mechanical property of resistance to bending when they are debonded [20]. Furthermore, some of these aesthetic brackets can combine both mechanical and chemical (silica coating) retention to increase bond strength [21]. In the present study, metal brackets retention system consists of a microetched mesh pad attached to the base; ceramic brackets achieve retention via their microcrystalline base with no chemical treatment. The differences in SBS between the two bracket types, which were evidently not dependent on the zirconia surface treatment applied, can be explained by both the low flexural properties of ceramic brackets, and the differences in the micromechanical retention system of their bases. A study conducted by Ansari et al. found that ceramic brackets with microcrystalline base achieved higher SBS when compared to other mechanical retention systems [22], which is in accordance with the high adhesion values obtained for ceramic brackets in the present study.

The surface treatments that provided the highest SBSs between zirconia and metallic brackets were the air-particle abrasion/femtosecond laser irradiation combination, femtosecond laser alone, and silica coating, with no significant differences between these groups (p>0.8). This fact is born out in SEM images, where notable roughness can be observed on the surfaces in these three groups (Fig 1), which would boost micromechanical retention and so bond strength [23, 24]. Both FS laser groups show deep grooves on the ceramic surfaces as observed in Fig 1, which leads to a greater penetration of the adhesive system hence increasing the adhesion strength. Although silica coating treatment creates similar surface irregularities to APA, SBS values are higher due to the silane agent application, which enhances the adhesion, being similar to FS laser groups.

Previous studies also obtained higher SBS values for specimens irradiated with femtosecond laser [12,13]. These authors used ceramic materials other than zirconia (feldspathic and lithium disilicate), the present study being the only one to have analyzed the SBS of orthodontic brackets bonded to femtosecond laser-treated zirconia surfaces. Neither silica coating nor the combination of laser with air-particle abrasion were tested by the authors cited above. ARI scores for the groups reporting higher SBS values were predominantly 2 and 3, in contrast to the control and APA groups. Erdur and Basciftci (2015) and Akpinar et al. (2015) also reported bond failures between the composite layer and the bracket for the femtosecond laser group [12,13].

Regarding the results obtained for ceramic brackets, no significant differences were found between air-particle abrasion and femtosecond (alone or combined) (p > 0.1). Furthermore, although no statistically significant differences were obtained between control group and FS laser alone in terms of shear bond strength, both groups showed different performance, as significant differences in terms of adhesive remnant were found. The results cannot be compared with any other study as no other work has assessed the SBS of ceramic brackets bonded to femtosecond-treated ceramic surfaces.

Irradiating the zirconia surfaces with femtosecond laser and applying laser after air-abrading the surface with Al_2_O_3_ obtained similar results. For this reason, the authors consider that the sandblasting procedure prior to laser application can be avoided, reducing costs, preparation time and patient discomfort.

In the present assay, the laser was set at a power output of 200mW, the chosen ablation pattern consisted of parallel lines separated by 60 μm and the samples were irradiated for 12 minutes. Due to the heterogeneity in terms of laser settings found in the published studies about femtosecond laser irradiation of ceramic surfaces [12–14], a pilot study was conducted by the authors (pending publication), to determine the ideal parameters, in which different power outputs and patterns were tested and irradiation time was optimized. The pilot study found these settings to be more effective in terms of SBS of brackets bonded to zirconia compared with the rest of the groups.

The present study demonstrated that femtosecond laser irradiation is an effective surface conditioning method for achieving good bond strength for brackets bonded to zirconia. This laser etches the surface gently but with precision, without producing mechanical degradation of the materials [25] and without raising the temperature of the irradiated surface [16], unlike other laser devices [26,27]. Furthermore, the present study found adhesive failure type 3 in most of the laser-treated samples, this conditioning technique being more conservative as the zirconia surface remains intact at debonding [28].

One limitation of the present study is that surface characterization (Raman analysis or X-ray Photoelectron Spectroscopy analyses) was not conducted. This would help to describe the interactions between the bonding surfaces. Another limitation is that only one type of metal and ceramic brackets were tested. Further studies should evaluate the performance of different brands of brackets on femtosecond laser-treated ceramic surfaces, since they are made of different materials and have different base designs.

Despite the advantages reported, femtosecond laser as a surface conditioning method has not yet been tested clinically due to the current costs and dimensions of the system. Further research is required before the technique may be introduced into clinical practice.

## Conclusions

- Within the limitations of this in-vitro study, femtosecond laser may be an effective surface-conditioning method for boosting the bond strength of ceramic and metallic orthodontic brackets bonded to zirconia.
- APA+FS laser irradiation was the most effective zirconia-conditioning technique when bonding both metallic and ceramic brackets, with no significant differences with silica coating and FS laser (metallic brackets subgroup) and with APA and FS laser (ceramic brackets subgroup).
- Ceramic brackets provide significantly higher adhesion strength to zirconia surfaces, regardless of the surface treatment method, compared to metallic brackets.
- Femtosecond laser irradiation is a conservative zirconia-conditioning technique since a great amount of the adhesive remains on the surface at debonding (ARI score 3)

## Supporting information

S1 File Study data. Shear bond strength values (MPa) for all specimens tested in the study.
(XLSX)

## References

[1] Zachrisson BU. Global trends and paradigm shifts in clinical orthodontics. World J Orthod. 2005;6:3–7 16958175

[2] Misch CE. Contemporary implant dentistry 3rd Ed. St. Louis: MOSBY; 2008.

[3] Gomes AL, Oyagüe RC, Lynch CD, Montero J, Albaladejo A. Influence of sandblasting granulometry and resin cement composition on microtensile bond strength to zirconia ceramic for dental prosthetic frameworks. J Dent. 2013;41:31–41 doi: 10.1016/j.jdent.2012.09.013 23022106 10.1016/j.jdent.2012.09.013

[4] Poosti M, Jahanbin A, Mahdavi P, Mehrnoush S. Porcelain conditioning with Nd:YAG and Er:YAG laser for bracket bonding in orthodontics. Lasers Med Sci. 2012; 27: 321–24 doi: 10.1007/s10103-010-0878-6 21243510 10.1007/s10103-010-0878-6

[5] Mosharraf R, Rismanchian M, Savabi O, Ashtiani AH. Influence of surface modification techniques on shear bond strength between different zirconia cores and veneering ceramics. J Adv Prosthodont. 2011;3:221–8 doi: 10.4047/jap.2011.3.4.221 22259706 10.4047/jap.2011.3.4.221 PMC3259448

[6] Spohr AM, Borges GA, Júnior LH, Mota EG, Oshima HM. Surface modification of In-Ceram Zirconia ceramic by Nd:YAG laser, Rocatec system, or aluminum oxide sandblasting and its bond strength to a resin cement. Photomed Laser Surg. 2008;26:203–8 doi: 10.1089/pho.2007.2130 18588435 10.1089/pho.2007.2130

[7] Usumez A, Hamdemirci N, Koroglu BY, Simsek I, Parlar O, Sari T. Bond strength of resin cement to zirconia ceramic with different surface treatments. Lasers Med Sci. 2013;28:259–66 doi: 10.1007/s10103-012-1136-x 22718473 10.1007/s10103-012-1136-x

[8] Cavalcanti AN, Foxton RM, Watson TF, Oliveira MT, Giannini M, Marchi GM. Bond strength of resin cements to a zirconia ceramic with different surface treatments. Oper Dent. 2009;34:280–7 doi: 10.2341/08-80 19544816 10.2341/08-80

[9] Akin H, Ozkurt Z, Kımalı O, Kazazoglu E, Ozdemir A. Shear bond strength of resin cement to zirconia ceramic after aluminium oxide sandblasting and various laser treatments. Photomed Laser Surg. 2011;29:797–802 doi: 10.1089/pho.2011.3039 22150095 10.1089/pho.2011.3039

[10] Paranhos MP, Burnett LH Jr, Magne P. Effect Of Nd:YAG laser and CO2 laser treatment on the resin bond strength to zirconia ceramic. Quintessence Int. 2011;42:79–89 21206937

[11] Gomes AL, Ramos JC, Santos-del Riego S, Montero J, Albaladejo A. Thermocycling effect on microshear bond strength to zirconia ceramic using Er:YAG and tribochemical silica coating as surface conditioning. Lasers Med Sci. 2015;30:787–95 doi: 10.1007/s10103-013-1433-z 24013623 10.1007/s10103-013-1433-z

[12] Erdur EA, Basciftci FA. Effect of Ti:sapphire laser on shear bond strength of orthodontic brackets to ceramic surfaces. Lasers Surg Med. 2015;47:512–9 doi: 10.1002/lsm.22371 25994849 10.1002/lsm.22371

[13] Akpinar YZ, Irgin C, Yavuz T, Aslan MA, Kilic HS, Usumez A. Effect of femtosecond laser treatment on the shear bond strength of a metal bracket to prepared porcelain surface. Photomed Laser Surg. 2015;33:206–12 doi: 10.1089/pho.2014.3791 25790117 10.1089/pho.2014.3791

[14] Vicente M, Gomes AL, Montero J, Rosel E, Seoane V, Albaladejo A. Influence of cyclic loading on the adhesive effectiveness of resin-zirconia interface after femtosecond laser irradiation and conventional surface treatments. Lasers Surg Med. 2016;48:36–44 doi: 10.1002/lsm.22442 26743446 10.1002/lsm.22442

[15] Vicente Prieto M, Caseiro Gomes AL, Montero Martín J, Alvarado Lorenzo A, Seoane Mato V, Albaladejo Martínez A. The Effect of Femtosecond Laser Treatment on the Effectiveness of Resin-Zirconia Adhesive: An In Vitro Study. J Lasers Med Sci. 2016;7:214–219 doi: 10.15171/jlms.2016.38 28491255 10.15171/jlms.2016.38 PMC5415497

[16] Varel H, Ashkenasi D, Rosenfeld A, Wähmer M, Campbell EEB. Micromachining of quartz with ultrashort laser pulses. Applied Physics A. 1997;65:367–73

[17] Kaygisiz E, Egilmez F, Ergun G, Yuksel S, Cekic-Nagas. Effect of different surface treatments on bond strength of recycled brackets to feldspathic porcelain. J Adhes Sci Technol. 2016;30:45–55.

[18] Kim NH, Kim YJ, Lee DY. Bond Strengths of Orthodontic Metal Brackets to Tribochemically Silica-coated Zirconia Surfaces Using Different 10-Methacryloyloxydecyl Dihydrogen Phosphate-containing Primers. J Adhes Dent. 2017;19:21–29 doi: 10.3290/j.jad.a37724 28195275 10.3290/j.jad.a37724

[19] Artun J, Bergland S. Clinical trials with crystal growth conditioning as an alternative to acid-etch enamel pretreatment. Am J Orthod. 1984 4;85:333–40. 6231863 10.1016/0002-9416(84)90190-8

[20] Verstrynge A, Ghesquiere A, Willems G. Clinical comparison of an adhesive precoated vs. an uncoated ceramic bracket system. Orthod Craniofac Res. 2004;7:15–20 doi: 10.1111/j.1601-6343.2004.0276n.x 14989750 10.1111/j.1601-6343.2004.0276n.x

[21] Mundstock KS, Sadowsky PL, Lacefield W, Bae S. An in vitro evaluation of a metal reinforced orthodontic ceramic bracket. Am J Orthod Dentofacial Orthop. 1999;116:635–41 10587597 10.1016/s0889-5406(99)70198-8

[22] Ansari MY, Agarwal DK, Gupta A, Bhattacharya P, Ansar J, Bhandari R. Shear Bond Strength of Ceramic Brackets with Different Base Designs: Comparative In-vitro Study. J Clin Diagn Res. 2016 11;10:ZC64–ZC68 doi: 10.7860/JCDR/2016/20624.8910 28050507 10.7860/JCDR/2016/20624.8910 PMC5198460

[23] Uehara K, Sakurai M. Bonding strength of adhesives and surface roughness of joined parts. J Mater Process Tech. 2002;127:178–181

[24] Moradabadi A, Roudsari SE, Yekta BE, Rahbar N. Effects of surface treatment on bond strength between dental resin agent and zirconia ceramic. Mater Sci Eng C Mater Biol Appl. 2014;1:311–7 10.1016/j.msec.2013.09.015 24268263

[25] Fiedler S, Irsig R, Tiggesbäumker J, Schuster C, Merschjann C, Rothe N, et al Machining of biocompatible ceramics with femtosecond laser pulses. Biomed Tech (Berl). 2013;58 (Suppl. 1) 10.1515/bmt-2013-4093 24042670

[26] Armengol V, Jean A, Marion D. Temperature rise during Er:YAG and Nd:YAP laser ablation of dentin. J Endod. 2000;26:138–41 doi: 10.1097/00004770-200003000-00002 11199705 10.1097/00004770-200003000-00002

[27] Malmström HS, McCormack SM, Fried D, Featherstone JD. Effect of CO_2_ laser on pulpal temperature and surface morphology: an in vitro study. J Dent. 2011;29:521–9 10.1016/s0300-5712(01)00028-8 11700201

[28] Smith GA, McInnes-Ledoux P, Ledoux WR, Weinberg R. Orthodontic bonding to porcelain-bond strength and refinishing. Am J Orthod Dentofacial Orthop. 1998;94:245–52 10.1016/0889-5406(88)90034-0 3046331

